# Rotatory instability of the knee after ACL tear and reconstruction

**DOI:** 10.1007/s10195-013-0254-y

**Published:** 2013-08-06

**Authors:** Andrea Ferretti, Edoardo Monaco, Antonio Vadalà

**Affiliations:** Orthopaedic Unit and Kirk Kilgour Sports Injury Center, Sant’andrea University Hospital, La Sapienza University, Via di Grottarossa, 1037, 00189 Rome, Italy

**Keywords:** ACL reconstruction, Single-bundle, Double-bundle, Navigator system, Rotatory instability

## Abstract

Although ACL reconstructions provide satisfactory clinical results nowadays, regardless of the type of graft or the surgical technique used (out-in vs in-out or single- vs double-bundle), the residual rotatory instability which is often detected at clinical follow-ups is still a matter of concern among surgeons. In this paper we try to analyze all the aspects which might contribute to this phenomenon by summarizing the biomechanical functions of the two bundles of the ACL, and by evaluating all the other factors strictly related to the rotatory instability of a reconstructed knee, such as the anatomical positioning of the single- or double-bundle new ACL, or the importance of a valid lateral compartment (LCL, ALTFL). Clinical, biomechanical and cadaver studies are discussed in order to contribute to better understanding of the origin of post-operative residual rotatory instability.

## Introduction

Anterior cruciate ligament (ACL) reconstruction is one of the most common orthopedic procedures. However, despite very satisfactory clinical outcomes [1, 2], the surgeon often detects the persistence of a certain rotatory instability, even in cases of reconstructions with no detectable intra- or post-operative complications, and regardless of the type of graft used [3]. The cause of this phenomenon is not completely understood yet, and the goal of many researchers is to be able to finally reconstruct a knee with the original rotational stability. In this paper all the aspects between rotatory instability and normal and torn ACL will be analyzed.

## Anatomy and function

The main function of the ACL is to control tibial anterior translation with a secondary effect on knee internal rotation [4, 5]. Both ACL functions are possible due to the presence of other articular structures which are linked to the ACL in maintaining knee stability. It is well documented how the ACL is made of two different bundles, the antero-medial (AM) and the postero-lateral (PL), with different specific functions, but working synergically so that they cannot be considered as separate structures.

One of the main aspects of ACL anatomy, in regard to surgical reconstruction, is the exact position of its femoral and tibial insertion. The femoral insertion has been the most commonly studied since the first attempts at open intra-articular reconstructions, because it is thought to be the most difficult to reproduce due to its very posterior positioning on the medial surface of the external femoral condyle [6]. It was common opinion that, despite the graft choice, the positioning of the graft could never be posterior enough, so many surgeons tried to perform an over the top technique; however, it is now appreciated that the native ACL femoral insertion site is located along osseous landmarks on the posterior aspect of the medial wall of the lateral femoral condyle, termed the lateral intercondylar and bifurcate ridges [7]. The lateral intercondylar ridge corresponds to the feature termed the resident ridge reported by Clancy et al. [8]. Identification of this ridge when present has been shown to be an accurate and reliable method of locating the native ACL insertion site and the true entry point of the femoral tunnel [9].

## Diagnosis

**Fig. 1:**
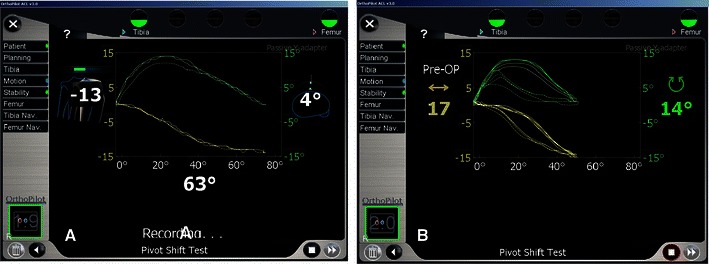
The pivot-shift phenomenon as detected by a navigator: tibial antero-posterior diagram and rotation in a knee with intact ACL (**a**) and in an ACL-deficient knee (**b**)

Diagnosis of ACL rupture is basically clinical, and is usually performed with the Lachman and the pivot-shift tests, whose sensibility and specificity have been widely documented. The pivot-shift test assesses both tibial translation and rotation, reproducing the most common traumatic mechanism of rupture of the ligament. Moreover, the pivot-shift test better correlates with a patient’s disability. For this reason it is thought to be the most reliable test for detecting rotatory instability of the knee following an ACL tear [10]. The pivot-shift is usually graded in three degrees (grade I: glide; grade II: jerk; grade III: subluxation), but it is highly dependent on the ability and experience of the operator. Since the importance of this test is in evaluating both ACL deficiency and the effects of different surgical techniques of reconstruction, many authors have proposed various methods of objectively evaluating and quantitatively measuring the test, such as the use of a navigation device (Fig. 1), mechanical or electromagnetic tool, or conventional radiological or magnetic resonance dynamic methods [11–17]. However, none of these methods have become widespread among surgeons and many of them remain operator-dependent. As a matter of fact the pivot-shift test is still the most popular test for assessing the rotatory instability of the knee after an ACL rupture and reconstruction.

## ACL tear and rotatory instability

The relationship between ACL lesions and the resulting rotatory instability of the knee has mainly been studied on cadaver knees. In fact, in in vivo studies where the instability was compared to the contralateral healthy knee, we can never assume that the lesion of the ACL was the only damage that occurred in the knee as a consequence of the initial trauma or as a result of progressive stretching of other structures due to the lack of the torn ACL. In a recent study we performed on ten cadaver knees, we evaluated internal rotator instability after sectioning either component of the ACL, showing that an isolated section of the PL bundle did not produce an increase in internal rotator instability; on the contrary, the section of the entire ACL did produce an increase in internal instability in nine cases. In our trials, only after a complete section of the entire ACL did we detect a + pivot-shift (glide) in three cases and a ++ pivot-shift (jerk) in seven cases; a +++ pivot-shift (subluxation) was never detected after isolated complete section of the ACL [18].

However, in regard to rotatory instability, ACL lesions, and the pivot-shift test, we should remember that the pivot-shift test assesses rotatory instability, and in particular, according to Hughston [19], the antero-lateral instability, whose origin is more complicated than a simple result of ACL lesion. Mueller et al. [20] and Feagin et al. [21] stated that even though the pivot-shift is related to an ACL tear, it is significantly increased by concomitant lesions of the external compartment and in particular of the “middle one-third of the lateral capsular ligament” (Hughston), also known as anterolateral femoral tibial ligament (ALFTL). Actually, in our study cited above on cadaver knees we detected a significant increase in rotatory instability along with a +++ pivot-shift in all the cases in which, after tearing the entire ACL, even a partial lesion (1 cm) of the ALFTL was performed. Even more recently, Mushal et al. [22] showed, also with the use of a navigation system, a significant increase of the anterior tibial translation in the external compartment during the pivot-shift maneuver in ACL deficient knees after external meniscectomy. The same group of researchers had previously shown that the anterior translation of the external part of the tibia was strictly correlated to the pivot-shift score [23]. A similar result was achieved by Diermann et al. [11] using a robotic/UFS testing system.

In conclusion, we can state that, while an isolated lesion of the PL bundle does not seem to increase internal rotation, lesion of the entire ACL produces a slight increase of internal rotation but, most of all, a different pattern of rotation whose fulcrum moves from the central pivot of the joint to the medial collateral ligament (MCL), with following increase of the anterior translation of the external tibial plateau. The pivot-shift test, in assessing this particular biomechanical pattern of instability, is related not only to the lesion of the ACL, but it is strongly influenced by associated lesions, in particular those of the lateral compartment (ALFTL, LCL, lateral meniscus).

## Effects of ACL reconstruction on rotatory instability

Even though ACL reconstruction is a very widely used operation with a very satisfactory success rate which allows the majority of active patients involved in sports activities to resume their pre-operative levels, the persistence of a certain rotatory joint laxity might produce subsequent meniscal or chondral damage leading to a clear degenerative arthritic disease [24]. Even though the pivot-shift assessment has always been part of the evaluation scoring scales used, it has often been underestimated; only within the last few years has this topic come back to scientific attention thanks to Freddi Fu’s studies on the functional anatomy of the ACL, on its two distinct bundles and, most of all, on failure of the single-bundle reconstruction to restore exact joint stability especially in regard to internal rotation. As a result, many surgeons, with the aim of improving their success rates, have in turn started using the double-bundle reconstruction of the ACL. In recent years, and in particular in the first years of the third millennium, many clinical and laboratory studies have been published on the advantages of reconstruction of the two bundles, thus supporting Fu’s theory [25–27]. In particular, clinical studies with a minimum follow-up of 2 years in which double-bundle reconstructions were used showed better results than the single-bundle, both in terms of knee stability and in terms of recurrence rate. Moreover, other studies, such as the one by Robinson et al. [28] on cadaver knees with the use of a navigation system, further provided objective data about how, in ACL reconstructions, the AM and PL bundles act differently in stabilizing the knee, particularly during the pivot-shift test, where the PL bundle is important in controlling not only anterior laxity toward knee extension, but also the rotational component. In net contrast with what was reported by the above mentioned authors, in a similar study we performed on cadaver knees with the use of a navigation system, we found that the further addition of the PL bundle to an AM single-bundle reconstruction did not provide any additional stability to the knee in regard to internal rotation. How might these different results be explained since the methodologies of the studies were similar? The explanation may likely be in the different surgical way of reconstructing the AM bundle: in fact, in Robinson’s study, the AM bundle was positioned slightly more anterior and vertical than in our study, whereas we tried to place the femoral insertion, approached with an out-in technique, more horizontal and as posterior as possible, thus in accordance with the actual anatomy of the ACL. This is a very important topic which deserves deeper examination [29]. Since the beginning of the reconstruction of the ACL with open techniques, it was mandatory for the surgeon to scrupulously respect the anatomy of the ACL with a very posterior positioning of the femoral insertion. Since this goal could not be reached by drilling the femoral condyle from the articular joint, surgeons started using the out-in technique. After a few years, with the advent of arthroscopic techniques, the majority of surgeons preferred to perform less invasive techniques (single-incision techniques), performing trans-tibial femoral tunnel drilling, often resulting in a non-anatomical positioning of the ACL. It seems as if, despite the advantages provided by the scope, the arthroscopists preferred mini-invasiveness over respect of the anatomy and function.

However, since rotatory instability is a complex phenomenon not simply dependent on the ACL rupture or the anatomical reconstruction, other hypotheses have been proposed to correct this type of instability. Among these, an important aspect is represented by the peripheral plasties [30, 31], whose biomechanical role in controlling rotational instability and the pivot-shift has been widely proven, even in recent studies published by our group: internal rotation was better restored in cases in which the anatomic ACL reconstruction was performed along with a peripheral plasty than in cases treated with a double-bundle technique [32]. More recently, Colombet et al. [33] did not reach the same conclusions as us: however, they did not put any tension on the lateral sling, thus losing a big part of the efficacy of the technique itself. Even more controversial is the clinical effectiveness of the peripheral plasties in long-term follow-up, even though Zaffagnini et al. [34], in assessing three groups of patients treated with hamstrings (STG), bone-patellar-tendon-bone (BPTB) and ST plus peripheral plasty, obtained the best results in the third group. In our experience we found peripheral plasty useful in patients with severe rotatory instability (+++ pivot-shift), in women and in cases of revision.

A complete lesion of the ACL often causes a rotatory instability of the knee detectable with the pivot-shift test. During this complex phenomenon the tibia tends to internally rotate with respect to the femur, thus changing its rotational fulcrum which moves medially closer to the MCL, with following anterior translation of the external tibial plateau. The severity of the pivot-shift, commonly scored in three degrees, essentially depends on the amount of constitutional tibial rotation and on the presence of concomitant associated lesions, such as the ALFTL and the external meniscus. In order to obtain the best rotatory stability, the ACL reconstruction must be performed accurately, reproducing its anatomical positions (either single- or double-bundle). Non-anatomical reconstructions are basically erroneous. Peripheral plasties may contribute to better control of rotator instability and may be indicated in selected types of patients.
